# Local immune and inflammatory endotypes of the nasal mucosa in allergic and non-allergic chronic rhinitis

**DOI:** 10.3389/fimmu.2026.1831730

**Published:** 2026-05-21

**Authors:** Zhancheng Ma, Cuiling Wu

**Affiliations:** 1 Department of Otorhinolaryngology-Head and Neck Surgery, the First Hospital of Jilin University, Changchun, Jilin, China; 2 Nursing College of Changchun Medical College of Higher Learning, Changchun, Jilin, China

**Keywords:** allergic rhinitis, inflammatory endotype, local immunity, nasal mucosa, non-allergic rhinitis

## Abstract

**Background:**

Allergic rhinitis (AR) and non-allergic rhinitis (NAR) are the main forms of chronic rhinitis with distinct pathogenesis. Endotype analysis based on nasal mucosal immune markers may facilitate precision medicine.

**Objective:**

To compare inflammatory profiles and identify endotypes in AR and NAR through local immune marker detection and correlation analysis.

**Methods:**

This retrospective study enrolled chronic rhinitis patients (January 2023–June 2025) divided into three groups: AR group [ARG, n=40, positive skin prick test and/or serum specific IgE (sIgE)], NAR group (NARG, n=40, negative allergy tests), and control group (CG, n=20). Primary measures: nasal eosinophil (EOS) and neutrophil (NEU) counts, sIgE and eosinophil cationic protein (ECP), Th2 cytokines, NEU-related mediators, and immune cell subsets.

**Results:**

ARG showed significantly higher EOS count, sIgE, ECP, interleukin-5 (IL-5), interleukin-13 (IL-13), and EOS% than NARG and CG. NARG exhibited significantly higher NEU count, interleukin-8 (IL-8), interleukin-1β (IL-1β), and NEU% (*P* < 0.01). Correlation analysis revealed moderate to strong positive correlations among Th2 markers in ARG and among NEU markers in NARG, with ECP also correlated to EOS in NARG (all *P* < 0.05).

**Conclusion:**

AR exhibits a Th2-dominant inflammatory network involving eosinophils, IgE, and Th2 cytokines rather than eosinophils alone, while NAR displays a NEU-mediated pattern with a subset showing eosinophilic activation. Multi-marker detection in nasal secretions effectively differentiates inflammatory endotypes in chronic rhinitis.

**Significance & innovation:**

This study identified a synergistic Th2 network in AR and a NEU-dominant pattern in NAR via nasal secretion analysis, providing evidence for endotype identification and precision therapy.

## 1 Introduction

Chronic rhinitis is one of the most prevalent conditions in otorhinolaryngology, affecting 10%-30% of the global population and substantially impairing quality of life and work productivity (1–3). Based on allergic mechanisms, chronic rhinitis is conventionally categorized into allergic rhinitis (AR) and non-allergic rhinitis (NAR) (4–6). AR represents a type I hypersensitivity reaction mediated by immunoglobulin E (IgE), characterized by Th2-dominant inflammation (7–9). Conversely, NAR encompasses a heterogeneous group of disorders, including vasomotor rhinitis, NAR with eosinophilia syndrome (NARES), and occupational rhinitis, whose complex and diverse pathogenic mechanisms remain incompletely understood (10–12). Recent advances in immunopathological research have revealed that conventional phenotyping based solely on clinical manifestations and allergy test results inadequately captures disease complexity. Patients presenting similar clinical symptoms may harbor distinct underlying immune-inflammatory mechanisms, defined as “endotypes.” Endotype classification, grounded in specific pathophysiological processes and molecular biomarkers, facilitates accurate diagnosis and individualized treatment. While Th2-skewed inflammation—characterized by elevated Th2 cytokines [interleukin-5 (IL-5) and interleukin-13 (IL-13)], eosinophil (EOS) infiltration, and local IgE production—is well-established in AR, the immunopathological features of NAR remain poorly defined (13–15). Some NAR patients may exhibit localized allergic responses, whereas others predominantly display neutrophil (NEU)-driven inflammation.

Nasal secretion analysis, a non-invasive approach for assessing local immune status, has garnered increasing attention (16). Unlike serum measurements, nasal secretions more directly reflect the mucosal inflammatory microenvironment, encompassing local IgE synthesis, cytokine profiles, and effector molecule release. Previous investigations have confirmed the presence of house dust mite-specific IgE (sIgE) and immunoglobulin A (IgA) in AR nasal secretions, correlating significantly with eosinophil cationic protein (ECP) levels (17–19). Additionally, elevated concentrations of various inflammatory mediators—including interleukin-1β (IL-1β), interleukin-6 (IL-6), and interleukin-8 (IL-8)—have been documented in nasal mucosa of adolescents with AR, associated with diminished quality of life (20–22). Nevertheless, comprehensive studies simultaneously measuring multiple inflammatory markers in nasal secretions to systematically compare local immune profiles between AR and NAR remain scarce.

Therefore, this retrospective cohort study was designed to collect nasal cytological features, allergic markers, Th2 cytokines, NEU-related mediators, and immune cell subsets from nasal secretions and brush samples. By systematically comparing local immune characteristics between AR and NAR patients and elucidating inflammatory network patterns through correlation analysis, we aim to identify distinct inflammatory endotypes, thereby providing theoretical foundations and actionable biomarkers for precise phenotyping and targeted therapeutic interventions in chronic rhinitis.

## 2 Materials and methods

### 2.1 Study population

**Figure 1:**
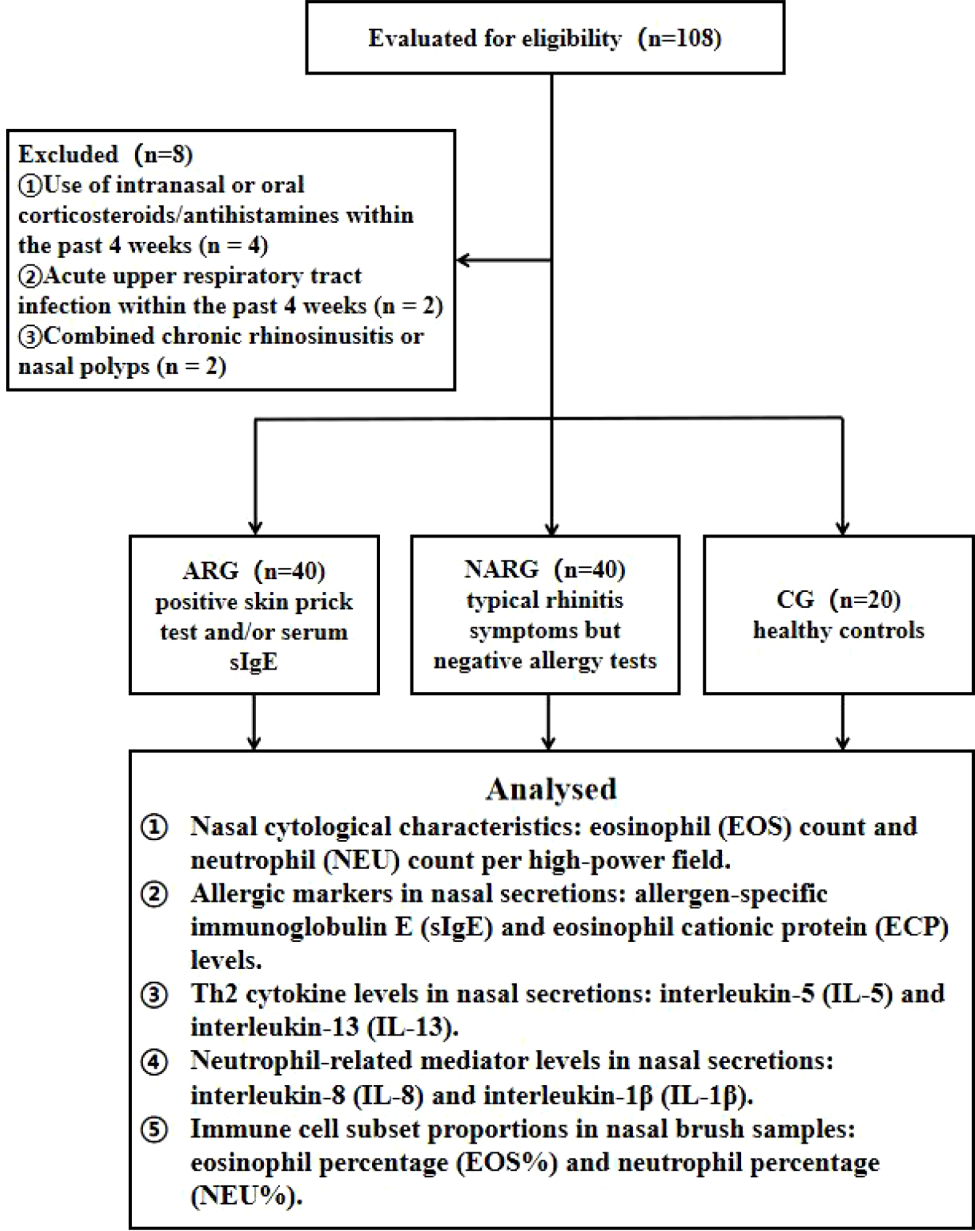
Research flowchart.

This single-center retrospective study enrolled patients with chronic rhinitis who visited the Department of Otorhinolaryngology at our hospital between January 2023 and June 2025. Clinical data and nasal secretion test results were extracted from the electronic medical record system. Inclusion criteria (23): ① Age 18–65 years; ② Diagnosed with AR according to the *Chinese Guideline for Diagnosis and Treatment of Allergic Rhinitis* (2022, revised edition) or presenting typical nasal symptoms with negative allergy tests; ③ Underwent nasal secretion collection and nasal brush cytology examination; ④ Complete clinical records. Exclusion criteria (24): ① Comorbid chronic sinusitis, nasal polyps, or severe nasal septum deviation; history of nasal surgery within the past 12 months or any sinus surgery; ② Acute respiratory infection/sinusitis within 4 weeks; ③ Use of intranasal/oral corticosteroids or antihistamines within 4 weeks; ④ Active comorbid allergic diseases such as asthma or atopic dermatitis; ⑤ Autoimmune diseases, immunodeficiency disorders, or ongoing immunomodulatory therapy; ⑥ Pregnancy or lactation; ⑦ Severe cardiac, pulmonary, hepatic, or renal dysfunction. A total of 80 patients were ultimately enrolled and divided into two groups based on allergy test results: AR group (ARG, n=40) and NAR group (NARG, n=40). Additionally, 20 healthy individuals were also enrolled as the control group (CG). The study flowchart is presented in **Figure 1**.

### 2.2 Ethical considerations

Approval was obtained from the First Hospital of Jilin University ethics committee (Approval No. ky2025121), and the study adhered to the Declaration of Helsinki and international ethical guidelines. Given the retrospective design using archived clinical data, the ethics committee waived the requirement for informed consent. The data were anonymized to protect participant privacy throughout the study.

### 2.3 Calculation of sample size

This retrospective case-control study referenced preliminary clinical data and literature, with estimated sIgE levels of approximately 8.5 IU/mL in the ARG, 0.5 IU/mL in the NARG, and 0.3 IU/mL in the control group (25). With α=0.05 (two-tailed), β=0.2 (power 80%), and balanced group allocation, PASS 15.0 software calculated a minimum required sample size of 32 patients per group. Considering potential incomplete clinical data or missing laboratory measurements inherent to retrospective studies, 88 patients were initially screened. After applying the inclusion and exclusion criteria, 80 patients remained, including 40 in the ARG and 40 in the NARG. Additionally, 20 healthy individuals were also enrolled as CG, resulting in a total sample size of 100 cases.

### 2.4 Observational parameters

Data for the following observational parameters were retrospectively extracted and collected through querying the hospital electronic medical record system, communication systems, and nursing records.

#### 2.4.1 Baseline data

Extract demographic information about the patient (age, sex, BMI); clinical features (family history of allergy, previous nasal surgery, alcohol consumption); comorbidities (asthma, atopic dermatitis, urticaria, drug allergy history, hypertension, diabetes); nasal symptom scores [total nasal symptom score (TNSS, four-item version), nasal congestion visual analogue scale (VAS)]; and nasal endoscopic findings (pale and edematous nasal mucosa, inferior turbinate hypertrophy).

#### 2.4.2 Nasal cytology

Nasal brush cytology was performed concurrently with nasal secretion collection. Brush samples were smeared onto slides and stained with Swiss-Giemsa stain. EOS and NEU counts were examined under light microscopy (400× magnification), with the mean value calculated from five randomly selected fields.

#### 2.4.3 Inflammatory markers in nasal secretions

sIgE and ECP levels were measured using the ImmunoCAP system. IL-5, IL-13, IL-8, and IL-1β concentrations were determined by commercial ELISA kits (R&D Systems, USA): human IL-5 (catalog #D5000, sensitivity 0.5 pg/mL), IL-13 (catalog #D1300, sensitivity 0.8 pg/mL), IL-8 (catalog #D8000C, sensitivity 1.0 pg/mL), and IL-1β (catalog #DLB50, sensitivity 0.3 pg/mL), following the manufacturer’s protocols.

#### 2.4.4 Immune cell subsets

Nasal brush samples were immediately placed in PBS. Cells were dispersed by gentle pipetting, filtered through a 70-μm cell strainer, and washed with cold PBS. Cell suspensions were incubated with fluorochrome-conjugated surface marker antibodies at 4 °C for 30 minutes in the dark. The following antibodies were used: anti-human CD15-FITC (clone HI98, BD Biosciences, USA) for neutrophils, and anti-human Siglec-8-PE (clone 7C9, BioLegend, USA) for eosinophils. After washing, EOS% and NEU% were determined by a CytoFLEX LX flow cytometer (Beckman Coulter, USA) equipped with 488 nm, 638 nm, and 405 nm lasers. Data were analyzed using FlowJo v10 software, and percentages were expressed relative to total CD45+ leukocytes (anti-CD45-APC-Cy7, clone 2D1, BD Biosciences).

### 2.5 Statistical analysis

The data were analyzed using SPSS 26.0 software. Data normality and variance homogeneity were tested by Shapiro-Wilk and Levene tests. Data that met the criteria of normal distribution and homogeneity of variance were represented as mean ± standard deviation. Comparisons between the three groups were performed using one-way ANOVA, and pairwise comparisons were corrected using the Bonferroni method for multiple comparisons. Count data were represented as counts (percentages), and comparisons between groups were conducted using chi-square tests or Fisher’s exact tests. Spearman correlation analysis was employed to evaluate associations among inflammatory markers within each group. All statistical tests were two-sided, and *P* < 0.05 was considered to indicate statistical significance.

## 3 Results

### 3.1 Baseline data comparison

As shown in **Table 1**, no significant differences were observed in age, sex, BMI, history of nasal surgery, alcohol consumption, or comorbidities including urticaria, drug allergy, hypertension, and diabetes (*P*>0.05). The ARG exhibited significantly higher proportions of family allergy history (*P* = 0.008), asthma (*P* = 0.035), and atopic dermatitis (*P* = 0.030) compared to NARG and CG. Regarding nasal symptom scores, TNSS and nasal congestion VAS were significantly elevated in ARG relative to NARG and CG (*P* < 0.001). Nasal endoscopic examination revealed significantly higher rates of pale and edematous nasal mucosa and inferior turbinate hypertrophy in ARG compared to NARG and CG (*P* < 0.001), confirming good comparability among groups.

**Table 1:** Baseline demographic and clinical characteristics.

Characteristic	ARG (n=40)	NARG (n=40)	CG (n=20)	*F/χ²*	*P*
Demographic characteristics					
Age ( $\bar{\text{x}}$ ± s, years)	35.25 ± 8.32	38.10 ± 8.70	35.10 ± 8.91	1.369	0.259
Male (n, %)	22 (55.00)	21 (52.50)	10 (50.00)	0.141	0.932
BMI ( $\bar{\text{x}}$ ± s, kg/m^2^)	23.18 ± 3.15	23.63 ± 3.39	22.95 ± 3.02	0.351	0.705
Clinical characteristics					
Family history of allergy (n, %)	18 (45.00)	7 (17.50)	3 (15.00)	9.598	0.008
Previous nasal surgery (n, %)	2 (5.00)	3 (7.50)	0 (0.00)	0.526	0.769
Alcohol consumption (n, %)	8 (20.00)	10 (25.00)	4 (20.00)	0.087	0.957
Comorbidities					
Asthma (n, %)	9 (22.50)	2 (5.00)	0 (0.00)	6.691	0.035
Atopic dermatitis (n, %)	8 (20.00)	1 (2.50)	0 (0.00)	7.021	0.030
Urticaria (n, %)	5 (12.50)	2 (5.00)	0 (0.00)	1.767	0.413
Drug allergy history (n, %)	4 (10.00)	1 (2.50)	0 (0.00)	1.579	0.454
Hypertension (n, %)	3 (7.50)	5 (12.50)	2 (10.00)	0.278	0.870
Diabetes (n, %)	1 (2.50)	2 (5.00)	2 (5.00)	0.130	0.937
Nasal symptom scores					
TNSS ( $\bar{\text{x}}$ ± s, points)	7.38 ± 2.17	6.13 ± 2.34	0.60 ± 0.50	76.677	< 0.001
Nasal congestion VAS ( $\bar{\text{x}}$ ± s, points)	6.83 ± 1.92	6.35 ± 2.01	0.55 ± 0.51	93.211	< 0.001
Nasal endoscopic findings					
Pale and edematous nasal mucosa (n, %)	32 (80.00)	18 (45.00)	0 (0.00)	34.800	< 0.001
Inferior turbinate hypertrophy (n, %)	28 (70.00)	24 (60.00)	0 (0.00)	27.885	< 0.001

BMI, Body Mass Index; TNSS, total nasal symptom score; VAS, visual analogue scale. Continuous variables (Age, BMI, TNSS, Nasal congestion VAS) were analyzed by one-way ANOVA. Categorical variables (Male, Family history of allergy, Previous nasal surgery, Alcohol consumption, Asthma, Atopic dermatitis, Urticaria, Drug allergy history, Hypertension, Diabetes, Pale and edematous nasal mucosa, Inferior turbinate hypertrophy) were analyzed by Chi-square test.

### 3.2 Nasal cytology

As shown in **Table 2**, significant differences among the three groups were observed in both EOS and NEU counts (both *P* < 0.001). Pairwise comparisons demonstrated significant differences between ARG and NARG, ARG and CG, and NARG and CG for both cell types (all *P* < 0.001). ARG was characterized by elevated EOS counts, whereas NARG predominantly exhibited increased NEU counts. These findings suggest that EOS and NEU counts differ significantly between ARG and NARG and may serve as potentially useful distinguishing parameters; their differential distribution may reflect distinct local inflammatory endotypes, providing preliminary cytological evidence for phenotyping. Nevertheless, the diagnostic performance of these parameters (e.g., sensitivity, specificity, cut-off values) requires formal evaluation using ROC analysis in future studies.

**Table 2:** Cytological characteristics of nasal mucosa.

Parameter	ARG (n=40)	NARG (n=40)	CG (n=20)	*F*	*P*1^1^	*P*2^2^	*P*3^2^	*P*4^2^
EOS (cells/HPF)	13.25 ± 3.47	3.53 ± 1.63	0.55 ± 0.51	241.014	< 0.001	< 0.001	< 0.001	< 0.001
NEU (cells/HPF)	5.13 ± 2.08	9.85 ± 3.61	1.75 ± 0.79	68.684	< 0.001	< 0.001	< 0.001	< 0.001

EOS, eosinophil; NEU, neutrophil; *P*1^1^, Overall comparison between the three groups, *p*-value (one-way ANOVA); ²Comparison between each pair: *P*2=ARG vs NARG, *P*3=ARG vs CG, *P*4=NARG vs CG. Same as above.

### 3.3 Allergic markers in nasal secretions

As shown in **Table 3**, significant differences among the three groups were observed in both sIgE and ECP levels (both *P* < 0.001). Pairwise comparisons revealed that sIgE and ECP levels were higher in ARG compared to NARG and CG (all *P* < 0.001), whereas no significant differences were found between NARG and CG for either marker (both *P*>0.05). These findings indicate that sIgE and ECP are significantly elevated in ARG compared to NARG and CG, suggesting their potential utility in reflecting local allergic inflammation.

**Table 3:** Comparison of allergic markers in nasal secretions.

Parameter	ARG (n=40)	NARG (n=40)	CG (n=20)	*F*	*P*1^1^	*P*2^2^	*P*3^2^	*P*4^2^
sIgE (IU/mL)	8.55 ± 3.82	0.51 ± 0.32	0.28 ± 0.16	133.432	< 0.001	< 0.001	< 0.001	0.936
ECP (μg/L)	188.32 ± 58.37	22.46 ± 10.31	17.36 ± 6.43	257.326	< 0.001	< 0.001	< 0.001	0.874

sIgE, secretory immunoglobulin E; ECP, eosinophil cationic protein.

### 3.4 Th2 cytokines in nasal secretions

As shown in **Table 4**, significant differences among the three groups were observed in both IL-5 and IL-13 levels (both *P* < 0.001). Pairwise comparisons demonstrated that IL-5 and IL-13 levels were higher in ARG compared to NARG and CG (all *P* < 0.001), whereas no significant differences were found between NARG and CG for either cytokine (both *P*>0.05). Th2 cytokines are markedly elevated in ARG, reflecting its Th2-dominant inflammatory phenotype, while NARG shows no evidence of Th2 activation, further supporting distinct immune-inflammatory mechanisms between these two forms of chronic rhinitis.

**Table 4:** Comparison of Th2 cytokines in nasal secretions.

Parameter	ARG (n=40)	NARG (n=40)	CG (n=20)	*F*	*P*1^1^	*P*2^2^	*P*3^2^	*P*4^2^
IL-5 (pg/mL)	38.94 ± 15.36	7.65 ± 2.94	5.17 ± 2.02	125.351	< 0.001	< 0.001	< 0.001	0.636
IL-13 (pg/mL)	42.69 ± 14.88	9.76 ± 4.06	6.32 ± 2.47	144.655	< 0.001	< 0.001	< 0.001	0.412

IL-5, interleukin-5; IL-13, interleukin-13.

### 3.5 NEU-related mediators

As shown in **Table 5**, significant differences among the three groups were observed in both IL-8 and IL-1β levels (both *P* < 0.001). Pairwise comparisons revealed that IL-8 and IL-1β levels were higher in NARG compared to ARG and CG. Additionally, both IL-8 and IL-1β levels were elevated in ARG compared to CG (all *P* < 0.001). These findings indicate that NEU-related mediators are markedly elevated in NARG, reflecting its NEU-dominant local inflammatory endotype. Although ARG showed modest elevations, its inflammatory profile is primarily Th2-driven, highlighting distinct inflammatory patterns between these two forms of chronic rhinitis.

**Table 5:** Comparison of NEU-related mediators.

Parameter	ARG (n=40)	NARG (n=40)	CG (n=20)	*F*	*P*1^1^	*P*2^2^	*P*3^2^	*P*4^2^
IL-8 (pg/mL)	69.46 ± 18.32	97.31 ± 23.64	42.14 ± 15.63	52.426	< 0.001	< 0.001	< 0.001	< 0.001
IL-1β (pg/mL)	12.77 ± 4.16	22.43 ± 5.91	6.49 ± 2.80	85.348	< 0.001	< 0.001	< 0.001	< 0.001

IL-8: interleukin-8; IL-1β: interleukin-1β.

### 3.6 Immune cell subsets

As shown in **Table 6**, significant differences among the three groups were observed in both EOS% and NEU% (both *P* < 0.001). Pairwise comparisons revealed that EOS% was higher in ARG compared to NARG and CG (both *P* < 0.001), whereas NEU% was highest in NARG, significantly exceeding values in both ARG and CG (both *P* < 0.001). Notably, EOS% in NARG was also higher than in CG (*P* < 0.001), reflecting the mixed phenotype of NARG with concurrent eosinophilic and neutrophilic elevation. In contrast, ARG exhibited predominantly elevated EOS%, characteristic of a Th2-dominant immunophenotype. Additionally, NEU% in ARG was higher than in CG (*P* < 0.05), suggesting that despite its Th2 predominance, ARG retains a degree of innate immune activation. These findings further substantiate fundamental differences in local immune cell composition between these two forms of chronic rhinitis.

**Table 6:** Comparison of immune cell subsets.

Parameter	ARG (n=40)	NARG (n=40)	CG (n=20)	*F*	*P*1^1^	*P*2^2^	*P*3^2^	*P*4^2^
EOS (%)	15.34 ± 3.92	3.37 ± 1.58	0.52 ± 0.24	285.830	< 0.001	< 0.001	< 0.001	< 0.001
NEU (%)	5.01 ± 1.82	11.54 ± 3.61	3.21 ± 1.04	92.984	< 0.001	< 0.001	0.035	< 0.001

### 3.7 Correlation analysis of nasal mucosal inflammatory markers

As shown in **Tables 7**–**9**, correlation analysis of nasal mucosal inflammatory markers revealed distinctly different patterns among the three groups. In ARG, Th2-related markers (sIgE, ECP, IL-5, IL-13, EOS) showed moderate to strong positive correlations (r=0.422-0.841, *P* < 0.01), forming a synergistic Th2 inflammatory network. Among NEU-related markers, IL-8 correlated with IL-1β (r=0.412, *P* < 0.01) and IL-1β correlated with NEU% (r=0.462, *P* < 0.01), while no significant correlation was observed between IL-8 and NEU% (r=0.130, *P>*0.05). In NARG, IL-8, IL-1β, and NEU% demonstrated moderate to strong positive correlations (r=0.441 - 0.722, *P* < 0.01), forming a NEU inflammatory network. Additionally, ECP correlated with EOS% (r=0.371, *P* < 0.05), IL-5 correlated with IL-13 (r=0.321, *P* < 0.05), and both correlated with EOS% (r=0.374 - 0.412, *P* < 0.05), reflecting eosinophilic activation characteristic of the NARES subtype within NARG. sIgE showed no significant correlations with any markers (*P*>0.05). In CG, only moderate correlations were observed between IL-8 and IL-1β (r=0.455, *P* < 0.05), IL-8 and NEU% (r=0.464, *P* < 0.05), and IL-1β and NEU% (r=0.485, *P* < 0.05), with no significant correlations among other markers, consistent with baseline characteristics of healthy individuals.

**Table 7:** Correlation matrix of inflammatory markers in nasal secretions among ARG.

Variable	sIgE	ECP	IL-5	IL-13	IL-8	IL-1β	EOS%	NEU%
sIgE	1	0.442**	0.621**	0.560**	0.086	-0.166	0.422**	-0.146
ECP		1	0.586**	0.630**	-0.055	-0.221	0.679**	0.060
IL-5			1	0.658**	-0.066	-0.093	0.753**	0.035
IL-13				1	-0.108	-0.180	0.841**	-0.045
IL-8					1	0.412**	-0.025	0.130
IL-1β						1	-0.108	0.462**
EOS%							1	0.098
NEU%								1

ARG (n=40). **P < 0.01.

**Table 8:** Correlation matrix of inflammatory markers in nasal secretions among NARG.

Variable	sIgE	ECP	IL-5	IL-13	IL-8	IL-1β	EOS%	NEU%
sIgE	1	0.031	-0.165	-0.206	0.129	0.055	-0.238	-0.017
ECP		1	0.108	-0.229	0.022	0.163	0.371*	-0.095
IL-5			1	0.321*	0.077	0.097	0.374*	0.156
IL-13				1	0.077	0.163	0.412**	-0.065
IL-8					1	0.703**	0.080	0.722**
IL-1β						1	0.263	0.441**
EOS%							1	-0.075
NEU%								1

NARG (n=40). *P < 0.05; **P < 0.01.

**Table 9:** Correlation matrix of inflammatory markers in nasal secretions among CG.

Variable	sIgE	ECP	IL-5	IL-13	IL-8	IL-1β	EOS%	NEU%
sIgE	1	-0.137	0.403	-0.109	-0.137	-0.030	0.260	-0.394
ECP		1	0.223	0.051	-0.108	-0.204	-0.168	-0.342
IL-5			1	0.269	-0.172	-0.163	0.087	-0.327
IL-13				1	0.114	-0.016	0.091	0.319
IL-8					1	0.455*	-0.119	0.464*
IL-1β						1	-0.183	0.485*
EOS%							1	-0.133
NEU%								1

CG (n=20). *P < 0.05.

## 4 Discussion

This study compared local immune profiles between AR and NAR patients through multi-marker detection in nasal samples, revealing distinct inflammatory networks via correlation analysis. ARG exhibited Th2-dominant inflammation characterized by a synergistic network of elevated sIgE, ECP, IL-5, IL-13, and EOS%, indicating that eosinophil elevation is part of a broader type 2 immune cascade rather than an isolated driving mechanism. Conversely, NARG displayed NEU-predominant inflammation, with increased IL-8, IL-1β, and NEU% showing moderate to strong positive intercorrelations. A subset of NARG patients demonstrated eosinophilic activation (ECP correlated with EOS%), indicating the NARES subtype. These findings support inflammatory endotyping in chronic rhinitis and identify potential biomarkers for targeted therapy.

ARG exhibited robust Th2-skewed inflammation. Compared to NARG and CG, ARG showed significantly elevated sIgE, ECP, IL-5, IL-13, and EOS%, with moderate to strong positive correlations among these markers, forming a highly synergistic Th2 network. These findings align with previous studies demonstrating higher EOS proportions in nasal lavage fluid from AR patients and positive correlations between total IgE and EOS (26–28). Other investigations have confirmed house dust mite-specific sIgE in AR nasal secretions, significantly correlated with ECP levels (29, 30). Furthermore, elevated IL-13 concentrations in AR nasal secretions have been associated with the degree of EOS infiltration.The underlying mechanism may involve allergen exposure triggering dendritic cells to present antigens and promote naive T cell differentiation toward the Th2 phenotype. Following sIgE binding to high-affinity receptors on mast cells, subsequent allergen exposure triggers degranulation, releasing effector molecules including ECP, thereby establishing a Th2-EOS-IgE positive feedback inflammatory loop. Among NEU-related markers, only IL-8 correlated with IL-1β and IL-1β correlated with NEU to a moderate degree, while IL-8 showed no significant correlation with NEU, indicating that the inflammatory pathway in ARG is relatively focused on the Th2 axis, with NEU inflammation remaining at a basal level. This aligns with the conventional understanding that AR is an IgE-mediated type I hypersensitivity reaction, with Th2 cell activation and EOS infiltration as core pathological mechanisms (31, 32). This relative specificity of the inflammatory pathway may be related to direct inhibitory effects of Th2 cytokines on NEU chemotactic factors.

The NARG exhibited marked inflammatory heterogeneity. First, NEU-related markers were significantly elevated compared to AR and control groups, with moderate to strong positive intercorrelations forming a distinct NEU inflammatory network. IL-8, the primary NEU chemoattractant, showed high concordance with NEU% (r=0.722), suggesting its role in driving NEU infiltration (33). IL-1β, a key proinflammatory cytokine in innate immunity, increased synergistically with IL-8 (r=0.703), potentially reflecting epithelial cell activation and innate immune pathway involvement in NAR (34). The underlying mechanism may involve environmental stimuli (pollutants, smoke, pathogen-associated molecular patterns) directly activating pattern recognition receptors on nasal epithelial cells in the absence of specific allergen stimulation, triggering proinflammatory cytokine release, subsequent NEU chemotaxis and activation, thereby establishing innate immune-mediated inflammatory responses. Additionally, activated NEUs can release IL-1β, further amplifying inflammatory signals and forming a NEU-epithelial positive feedback loop. Second, ECP correlated positively with EOS% in NAR (r=0.371), with EOS% (3.37%) significantly exceeding control values (0.52%), indicating EOS activation in a subset of NAR patients. This feature aligns partially with NARES (30, 35). Third, sIgE showed no significant correlations with any markers in NAR, and Th2 cytokines demonstrated no associations with NEU markers, indicating absence of Th2-skewed inflammation in NAR overall. This dissociation between Th2 and NEU pathways suggests that inflammatory endotypes in NAR may be driven by independent immune mechanisms: NEU-predominant NAR primarily involves innate immune pathway activation, while the NARES subtype involves localized type 2 inflammation, with these mechanisms operating relatively independently without interference.

Comparison of correlation matrices across groups revealed distinct inflammatory endotype patterns. In ARG, correlation coefficients among Th2 network markers ranged from 0.6-0.8, forming a tightly integrated “Th2 inflammatory module.” In NARG, the NEU network was similarly robust, with positive correlations between IL-8 and IL-1β, and between IL-1β and NEU. In CG, only moderate correlations emerged between IL-8, IL-1β, and NEU%, while other markers remained largely independent. This “pathological network reinforcement” suggests inflammatory endotypes in chronic rhinitis manifest as synergistic clusters of interrelated markers, rather than isolated elevations of single parameters (36). The underlying mechanism may involve the positive feedback regulation of inflammatory pathways: Under pathological conditions, the initial trigger factors (allergens or environmental stimuli) activate key transcription factors, leading to the coordinated expression of a series of genes related to the same pathway. Additionally, there is a mutual induction effect between effector molecules within the pathway, resulting in a cascading amplification effect. Furthermore, cytokines and effector molecules within the same inflammatory pathway are often driven by common upstream signals and are functionally interdependent, thus exhibiting a highly coordinated network structure in correlation analyses. In contrast, under healthy conditions, there is no sustained inflammatory driving signal, and each indicator maintains only baseline physiological fluctuations, resulting in relatively weak correlations. From a clinical translational perspective, this study holds significance. Firstly, nasal mucus testing, as a non-invasive method for assessing local immunity, can simultaneously detect multiple inflammatory markers, providing a “inflammatory fingerprint” for clinical use. Secondly, the combined detection of sIgE, ECP, IL-5, and IL-13 may help identify Th2-type inflammatory profiles, offering insights for the clinical application of biologics targeting IL-4/IL-13 and IL-5. Thirdly, the combined detection of IL-8, IL-1β, and NEU% may aid in identifying NEU-type NAR, providing a basis for exploring therapies targeting NEU recruitment pathways. Fourthly, the correlation between ECP and EOS% may assist in identifying NARES subtypes and preventing misdiagnosis with other types of NAR.

This study has several limitations. First, as a single-center retrospective study with relatively limited sample sizes, selection bias cannot be excluded, and generalization of findings warrants caution. Second, nasal allergen provocation tests were not performed, precluding complete exclusion of local AR cases potentially misclassified into the NARG. Third, no definitive diagnostic threshold for NARES was established, necessitating further refinement of subtype analysis in future investigations. Based on these findings, subsequent studies may explore multi-marker diagnostic models using nasal secretions and evaluate associations with symptom scores and quality of life measures. Furthermore, specific therapeutic options for NEU-predominant NAR remain limited. Drawing upon anti-inflammatory drug development strategies, future research may investigate novel therapeutic approaches targeting relevant inflammatory pathways (37).

## 5 Conclusion

In summary, AR and NAR exhibit distinct local immune profiles and inflammatory endotypes. AR is characterized by Th2-dominant inflammation, with sIgE, ECP, IL-5, IL-13, and EOS% forming a synergistic type 2 immune network. NAR predominantly displays NEU-mediated inflammation, with IL-8, IL-1β, and NEU% constituting a NEU-centered network. Multi-marker detection in nasal secretions combined with correlation network analysis may facilitate differentiation of inflammatory endotypes in chronic rhinitis, providing a preliminary reference for precise phenotyping and individualized therapeutic strategies. Formal diagnostic accuracy studies are needed before any single parameter can be recommended as a clinical diagnostic marker.

## Data Availability

The original contributions presented in the study are included in the article/supplementary material. Further inquiries can be directed to the corresponding author.
